# Characterization and phylogenetic analysis of the complete mitochondrial genome of *Cotylorhiza tuberculata* assembled using next-generation sequencing

**DOI:** 10.1080/23802359.2024.2406928

**Published:** 2024-09-25

**Authors:** Hui Jiang, Wangxiao Xia, Shaoxia Pu, Yanhua Su, Anzhu Zhu, Hongye Zhao, Hongjiang Wei, Yaowen Liu

**Affiliations:** a College of Life Science, Hainan Normal University, Haikou, China; b Shaanxi Key Laboratory of Brain Disorders, Institute of Basic Translational Medicine, Xi’an Medical University, Xi’an, China; c Yunnan Province Key Laboratory for Porcine Gene Editing and Xenotransplantation, Yunnan Agricultural University, Kunming, China; d Yunnan Province Xenotransplantation Research Engineering Center, Yunnan Agricultural University, Kunming, China; e Dongguan Xiangshi Zoo, Dongguan, China

**Keywords:** *Cotylorhiza tuberculata*, mitochondrial genome, next-generation sequencing, phylogenetic analysis

## Abstract

In this study, the complete mitochondrial genome (mitogenome) of *Cotylorhiza tuberculata* (Scyphozoa; Rhizostomeae; Cepheidae) was assembled by the next-generation sequencing data. The complete mitogenome spanned 16,590 bp and contained 14 protein-coding genes, two transfer RNA genes, and two ribosomal RNA genes. Total AT% content was 67.7%, comprising A 30.22%, C 16.16%, G 17.05%, and T 36.56%. The gene arrangement exhibited consistency with the known mitogenomes of other jellyfish species. Furthermore, the phylogenetic relationship of *C. tuberculata* was investigated based on analysis of the 13 common protein-coding genes. Results indicated a close relationship between *C. tuberculata* and both *Cassiopea xamachana* and *Cassiopea andromeda*. These findings provide a valuable reference for advancing understanding of the phylogenetic relationships, taxonomic classification, and phylogeography of jellyfish species.

## 1. Introduction

As a large marine jellyfish of Scyphozoa, *Cotylorhiza tuberculata* has an umbrella diameter of generally 35 centimeters and is mainly distributed in the Mediterranean, Aegean, and Adriatic seas (Kikinger 1992; İşinibilir et al. 2020). The distinctive egg yolk-like appearance of *C. tuberculata* in its adult form has led to its informal reference as the ‘fried egg jellyfish’ or ‘Medusa’, and it has been widely introduced in various countries for ornamental purposes (Kikinger 1992; Leone et al. 2013). Although *C. tuberculata* exhibits lower toxicity, it can still cause discomfort in people following contact (Carli et al. 1994; Riccio et al. 2022). Additionally, these jellyfish tend to congregate in substantial numbers during their reproductive season, adversely impacting bathing, fisheries and tourism (Cortés-Lara et al. 2015). At present, there has been some research on the growth and living habits of *Cotylorhiza tuberculata* (Kikinger 1992; İşinibilir et al. 2020), but there is still a lack of research data on its molecular characteristics and molecular biology aspects. We sequenced the mitochondrial genome of *Cotylorhiza tuberculata* and constructed a phylogenetic tree, aiming to provide basic information for the phylogenetic study of the genus *Cotylorhiza* and the evolutionary biology of jellyfish.

## 2. Materials and methods

**Figure 1.**
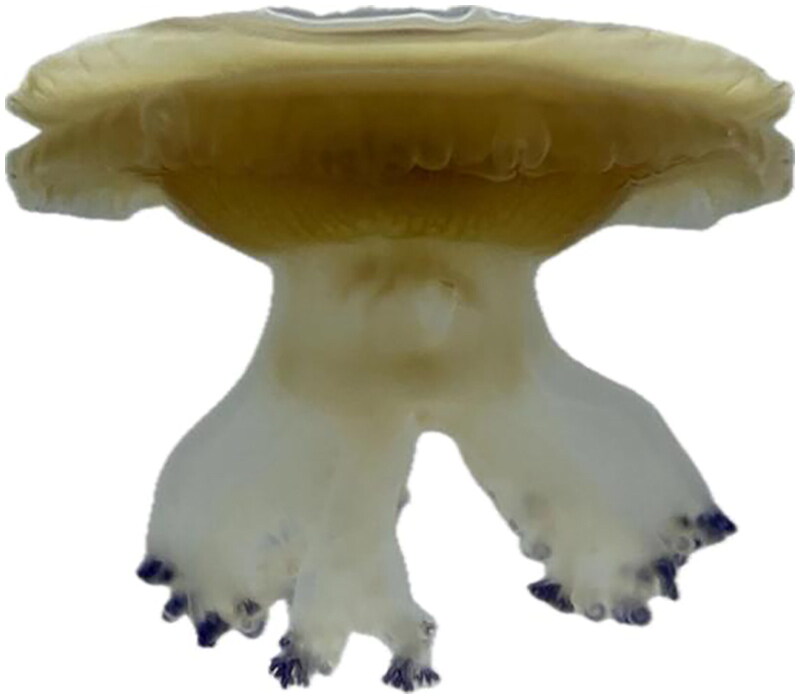
Reference image of *C. tuberculata*. Image was taken by LYW on 1 may 2023 at the biological laboratory of the College of Veterinary Medicine, Yunnan Agricultural University, China.

*Tuberculata* sample was collected from Xiamen, China (118°04'E, 24°26'N), and stored at the College of Veterinary Medicine, Yunnan Agricultural University, under voucher number Ctuberculata 2023-1 (Figure 1). After identifying the morphological features and molecular data of the specimen, genomic DNA was extracted using a Qiagen Blood & Cell Culture DNA Mini Kit. Then, short paired-end libraries were constructed according to the Illumina protocol and sequenced using the HiSeq X-Ten PE150 platform. Mitogenome assembly was performed using MitoZ software with default parameters (Meng et al. 2019). Gene prediction and annotation of the mitogenome were carried out using the MITOS web server (Bernt et al. 2013) and tRNAscan SE search server (Chan and Lowe 2019). The complete mitogenome assembly of *C. tuberculata* along with the annotation results were publicly released in GenBank (Accession number: OQ850984).

To determine the phylogenetic position of *C. tuberculata* among jellyfish, we downloaded 13 protein coding gene sequences of 15 Loricariidae and one outgroup (*Hydrozoanthus gracilis*) species from NCBI. 13 protein coding gene sequences shared by all species were aligned using ClustalW (Thompson et al. 1994) in BioEdit (Hall 1999). The neighbor-joining (NJ) phylogenetic tree was constructed using Mega7 with the 10,000 bootstraps (Kumar et al. 2016). The maximum-likelihood (ML) analysis (bootstrap test with 100 replications) was performed in MEGA X (Kumar et al. Citation2018) using GTR (General Time Reversible) + G model determined using the find Best DNA/Protein Models tool (embedded in MEGA X).

## 3. Results

**Figure 2.**
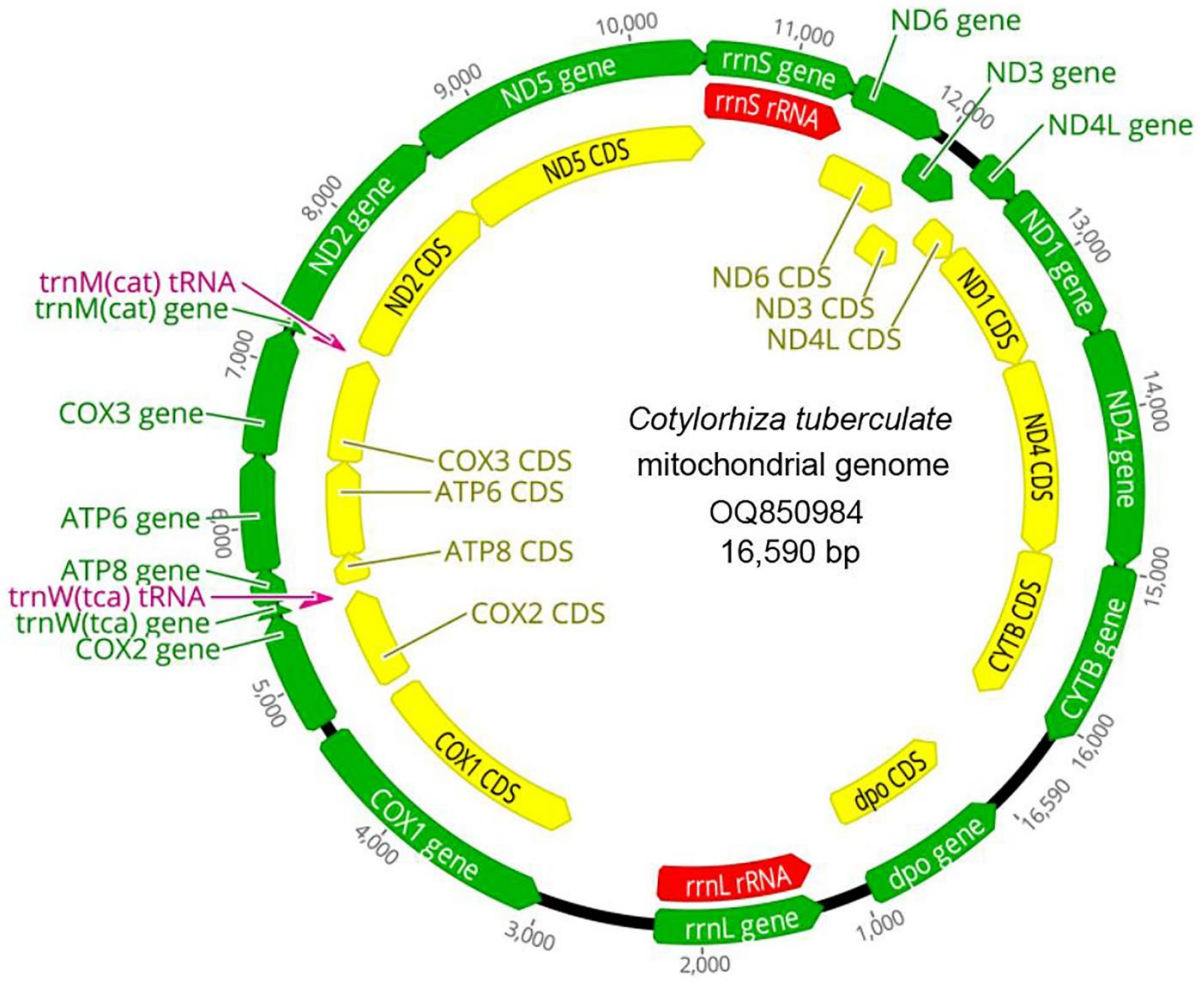
Circular gene map of *C. tuberculata* mitogenome.

The mitogenome of *C. tuberculata* spans 16,590 bp in length and contains 14 protein-coding genes, two transfer RNA (tRNA) genes (trna-W-TCA and trna-M-CAT), and two ribosomal RNA (rRNA) genes (12S and 16S rRNA) (Figure 2). This gene composition and arrangement align with those found in other jellyfish species (Li et al. 2016; Lisenkova et al. 2017; Karagozlu et al. 2019; Fang et al. 2022). Nearly all protein-coding genes were encoded by the H chain, except for 16S rRNA and *COX1* encoded on the L chain. Regarding the start codons, two protein-coding genes (*ND3* and *ND5*) started with GTG, while the remainder started with ATG. However, in terms of stop codons, three protein-coding genes (*COX2*, *CYTB*, and *ND6*) ended with TAG, while the others ended with TAA. The mitogenome exhibited significant AT bias, 66.79% AT and 33.21% GC, with a specific composition of A 30.22%, C 16.16%, G 17.05%, and T 36.56%. The 14 protein-coding genes encoded 3,997 amino acids, encompassing 11,991 bp and accounting for 72.28% of the entire mitogenome. The 12S rRNA has a predicted length of 934 bp between *ND5* and *ND6* while the 16S rRNA located between dpo and *COX1* has a predicted length of 1,731 bp.

**Figure 3.**
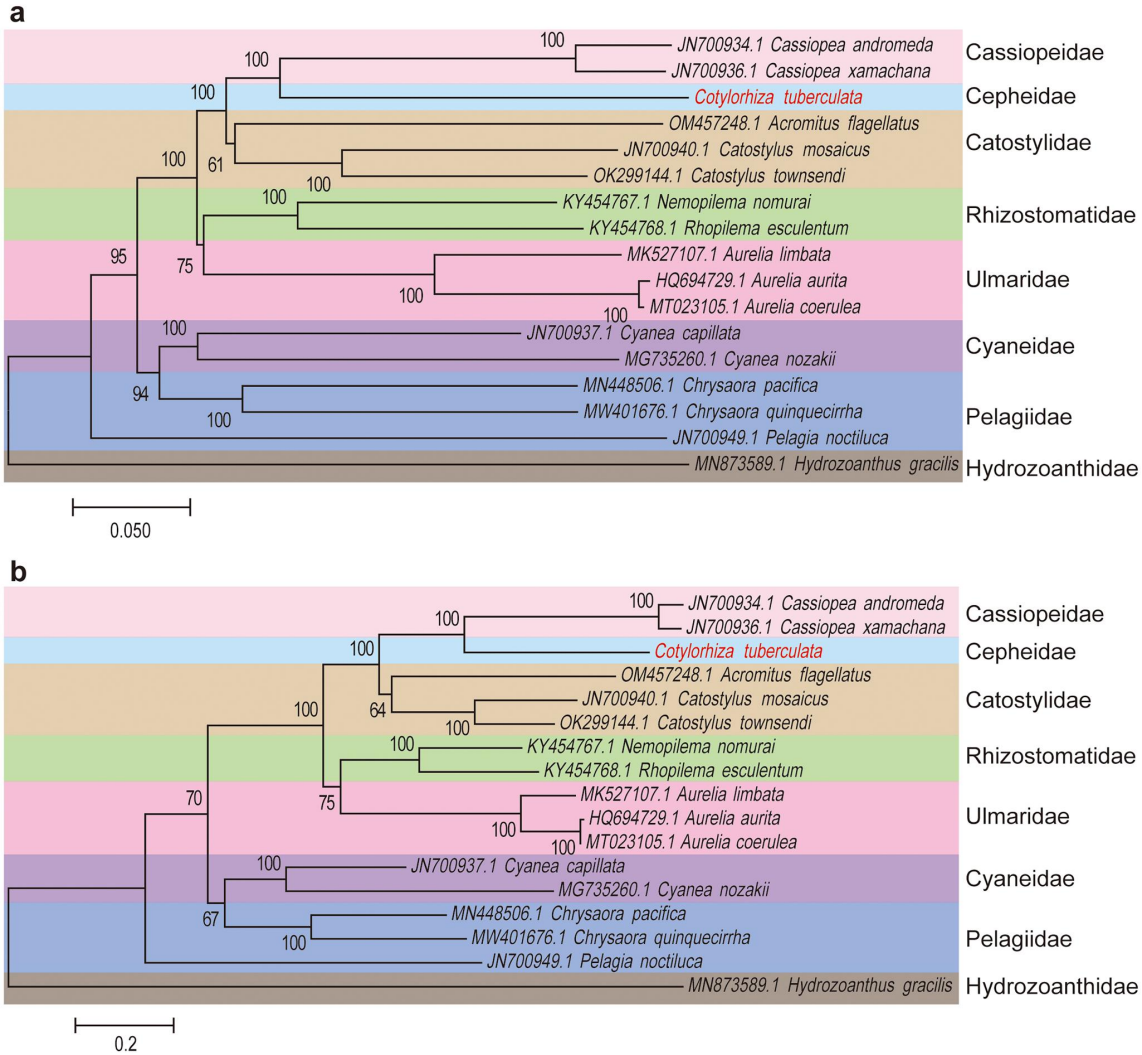
Phylogenetic trees of 16 Loricariidae and one outgroup (*Hydrozoanthus gracilis*) species based on 13 protein-coding genes. (a) The phylogenetic tree is constructed by neighbor-joining (NJ) method. (b) The phylogenetic tree is constructed by maximum-likelihood (ML) method. Numbers on each node are bootstrap probabilities. Number before species name is the GenBank accession number. Black dots indicate species in this study. The following sequences were used: *Cassiopea andromeda* JN700934.1 (Kayal et al. 2012), *Cassiopea xamachana* JN700936.1 (Kayal et al. 2012), *Cyanea capillata* JN700937.1 (Kayal et al. 2012), *Catostylus mosaicus* JN700940.1 (Kayal et al. 2012), *pelagia noctiluca* JN700949.1 (Kayal et al. 2012), *acromitus flagellates* OM457248.1 (Lin et al. 2022), *Catostylus townsendi* OK299144.1 (unpublished), *nemopilema nomurai* KY454767.1 (Wang and Sun 2017), *rhopilema esculentum* KY454768.1 (Wang and Sun 2017), *Aurelia limbata* MK527107.1 (Karagozlu et al. 2019), *Aurelia aurita* HQ694729.1 (Park et al. 2012), *Aurelia coerulea* MT023105.1 (Seo et al. 2020), *Cyanea nozakii* MG735260.1 (Karagozlu et al. 2018), *Chrysaora pacifica* MN448506.1 (Wang and Yin 2020), *Chrysaora quinquecirrha* MW401676.1 (unpublished), *Hydrozoanthus gracilis* MN873589.1 (Poliseno et al. 2020).

We conducted a comprehensive phylogenetic analysis of *C. tuberculata* and 15 other jellyfish species by constructing NJ (Figure 3(a)) and ML (Figure 3(b)) phylogenetic trees. The results showed that these 16 Loricariidae species formed 7 family branches, and *C. tuberculata* and two species (*Cassiopea andromeda* and *C. xamachana*) from the Cassiopeidae clustered into one branch (Figure 3). This study provides valuable insights into the evolutionary history of these jellyfish species and their shared genetic heritage.

## 4. Discussion and conclusions

In this study, the mitogenome of *C. tuberculata*, as described in this study, exhibits a typical gene composition and arrangement that is conserved among jellyfish species (Park et al. 2012; Kayal et al. 2012; Wang and Sun 2017; Karagozlu et al. 2019; Seo et al. 2020; Wang and Yin 2020; Lin et al. 2022). The presence of 14 protein-coding genes, two tRNA genes, and two rRNA genes is consistent with the mitochondrial genomes of some marine invertebrates (Cong et al. 2020; Xia et al. 2023). The finding that nearly all protein-coding genes are encoded by the H chain, with exceptions of 16S rRNA and *COX1* encoded on the L chain. This distribution of genes across the mitochondrial DNA may influence the replication and transcription processes, which are crucial for mitochondrial function (Tzagoloff and Dieckmann 2021). The observation of codon usage preferences, with GTG as the start codon for two genes and TAG as the stop codon for three genes, is intriguing. These preferences may reflect the translational machinery specific to jellyfish mitochondria, which could have evolved to optimize protein synthesis under the unique conditions of the jellyfish cellular environment (Parvathy et al. 2022). The finding that the 14 protein-coding genes encode 3,997 amino acids, accounting for 72.28% of the entire mitogenome, underscores the protein-coding capacity of the mitochondrial genome. This suggests that the mitochondria of *C. tuberculata* play a significant role in protein synthesis and, consequently, in jellyfish biology (Kummer and Ban 2021). Our findings revealed a strong phylogenetic affinity between *C. tuberculata* and two species of *Cassiopea*, namely *Cassiopea andromeda* and *Cassiopea xamachana*. This is significant as it provides valuable insights into the evolutionary history of these species and their shared genetic heritage. Overall, this study provides a comprehensive characterization of the mitogenome of *C. tuberculata*, revealing conserved features among jellyfish mitochondrial genomes, and highlights the importance of phylogenetic analysis in understanding species relationships and evolutionary patterns within jellyfish. The findings contribute to our understanding of mitochondrial genomics and evolution in jellyfish and have implications for future studies on jellyfish biology, including mitochondrial function, protein synthesis, and phylogeny.

## Supplementary Material

tuberculate_depth_supplemental Figure1.jpg

## Data Availability

The genome sequence data that support the findings of this study are openly available in GenBank of NCBI at [https://www.ncbi.nlm.nih.gov] (https://www.ncbi.nlm.nih.gov/) under the accession no. OQ850984.1. The associated BioProject, SRA, and Bio-Sample numbers are PRJNA992204, SRS18206865, and SAMN36344732 respectively.
