# Supplementary figures and images for: Characterization and phylogenetic analysis of the complete mitochondrial genome of Cotylorhiza tuberculata assembled using next-generation sequencing
Source: Mitochondrial DNA B Resour. 2024 Sep 25;9(9):1268–72. doi: 10.1080/23802359.2024.2406928 (PMC11425685; doi:10.1080/23802359.2024.2406928)

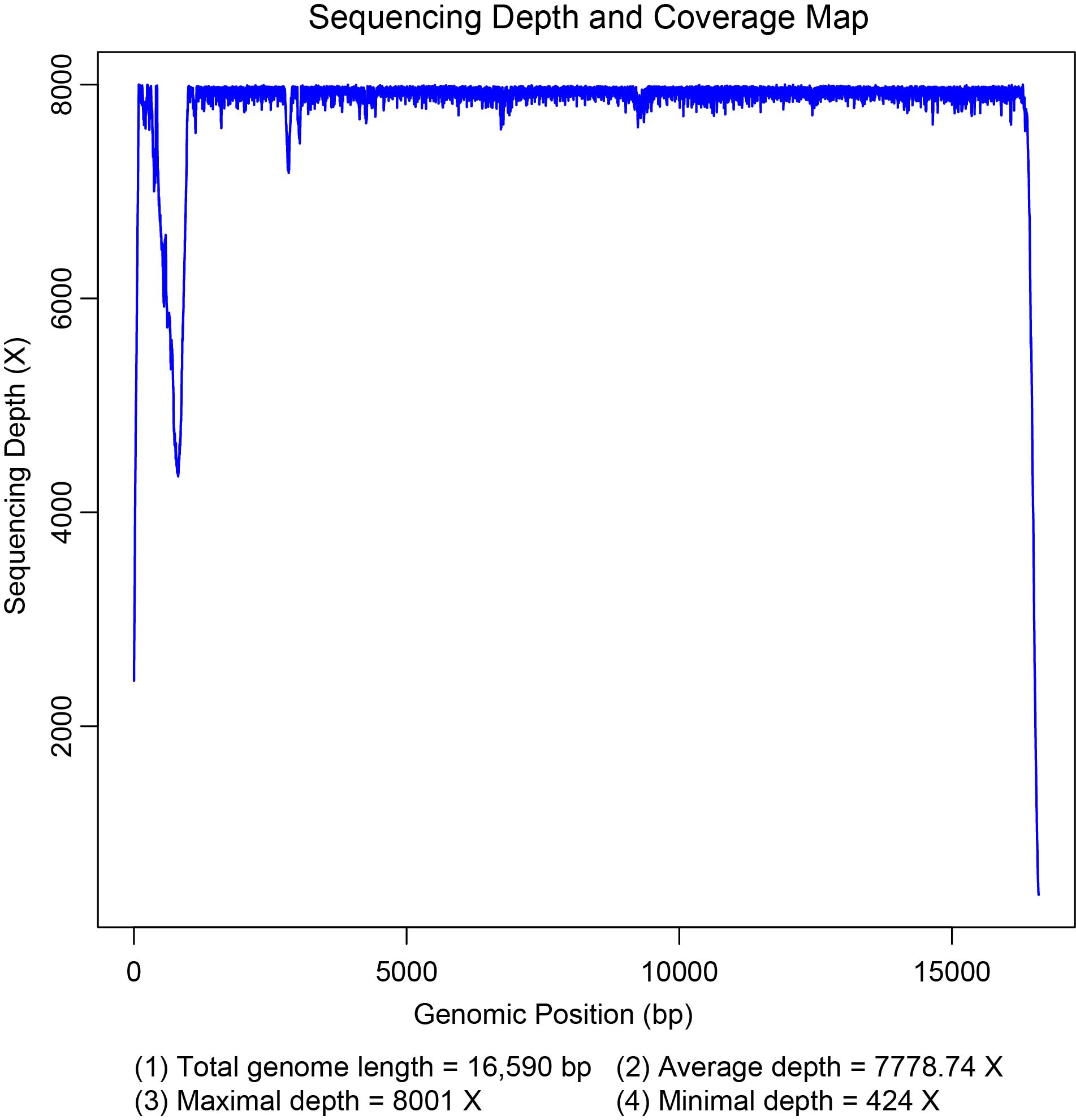

Supplement: tuberculate_depth_supplemental Figure1.jpg [file TMDN_A_2406928_SM0837.jpg]
